# Free Training in Midwifery

**Published:** 1888-03-24

**Authors:** Nurse Dore

**Affiliations:** Chapel-en-le-Frith, Derbyshire


### FREE TRAINING IN MIDWIFERY.

Would you be so very kind as to inquire through your valuable paper, The Hospital, if it is possible for a medical and surgical nurse to get midwifery training without paying. If so, where? Nurse Dore.

Chapel-en-le-Frith, Derbyshire.

